# Epithelial genetic muscarinic receptor 3 ablation induces sex-specific modulation of colonic intestinal progenitor cells and response to intestinal injury

**DOI:** 10.1093/ecco-jcc/jjaf038

**Published:** 2025-03-07

**Authors:** Mohab Ragab, Jessica Wieland, Caroline Waldherr Avila de Melo, Tatiana Agibalova, Anastasia Ermolova, Niklas Durner, Anneke Hempel, Fabian Heindl, H Carlo Maurer, Katja Steiger, Klaus-Peter Janssen, Markus Tschurtschenthaler, Timothy C Wang, Michael Quante, Roland M Schmid, Moritz Middelhoff

**Affiliations:** Department of Internal Medicine II, TUM University Hospital, Klinikum rechts der Isar, TUM School of Medicine and Health, Technical University of Munich, Munich, Germany; Department of Internal Medicine II, TUM University Hospital, Klinikum rechts der Isar, TUM School of Medicine and Health, Technical University of Munich, Munich, Germany; Department of Internal Medicine II, TUM University Hospital, Klinikum rechts der Isar, TUM School of Medicine and Health, Technical University of Munich, Munich, Germany; Department of Internal Medicine II, TUM University Hospital, Klinikum rechts der Isar, TUM School of Medicine and Health, Technical University of Munich, Munich, Germany; Department of Internal Medicine II, TUM University Hospital, Klinikum rechts der Isar, TUM School of Medicine and Health, Technical University of Munich, Munich, Germany; Department of Internal Medicine II, TUM University Hospital, Klinikum rechts der Isar, TUM School of Medicine and Health, Technical University of Munich, Munich, Germany; Department of Internal Medicine II, TUM University Hospital, Klinikum rechts der Isar, TUM School of Medicine and Health, Technical University of Munich, Munich, Germany; Department of Internal Medicine II, TUM University Hospital, Klinikum rechts der Isar, TUM School of Medicine and Health, Technical University of Munich, Munich, Germany; Department of Internal Medicine II, TUM University Hospital, Klinikum rechts der Isar, TUM School of Medicine and Health, Technical University of Munich, Munich, Germany; Institute of Pathology, TUM School of Medicine and Health, Technical University of Munich, Munich, Germany; Department of Surgery, TUM University Hospital, Klinikum rechts der Isar, TUM School of Medicine and Health, Technical University of Munich, Munich, Germany; Division of Translational Cancer Research, German Cancer Research Center (DKFZ) and German Cancer Consortium (DKTK), Heidelberg, Germany; Chair of Translational Cancer Research and Institute of Experimental Cancer Therapy, TUM University Hospital, Klinikum rechts der Isar, TUM School of Medicine and Health, Technical University of Munich, Munich, Germany; Center for Translational Cancer Research (TranslaTUM), TUM School of Medicine and Health, Technical University of Munich, Munich, Germany; Division of Digestive and Liver Diseases, Department of Medicine, Columbia University Medical Center, New York, NY, United States; Department of Medicine II (Gastroenterology, Hepatology, Endocrinology, and Infectious Diseases), Freiburg University Medical Center, Faculty of Medicine, University of Freiburg, Freiburg, Germany; Department of Internal Medicine II, TUM University Hospital, Klinikum rechts der Isar, TUM School of Medicine and Health, Technical University of Munich, Munich, Germany; Department of Internal Medicine II, TUM University Hospital, Klinikum rechts der Isar, TUM School of Medicine and Health, Technical University of Munich, Munich, Germany

**Keywords:** epithelial muscarinic receptor 3, colonic progenitor cells, colitis

## Abstract

**Background & Aims:**

Epithelial muscarinic acetylcholine receptor subtype 3 (M3R) signaling modulates intestinal stem and progenitor cell function, yet its impact on colonic homeostasis remains unclear. Hence, this study explores the sex-specific effects of epithelial genetic M3R ablation and muscarinic receptor agonism on murine colonic Lgr5-EGFP+ progenitor cells and epithelial homeostasis.

**Methods:**

Genetic ablation of M3R was achieved using Vil-Cre × M3R fl/fl mice. The effects on *Lgr5*-expressing progenitor cells, epithelial homeostasis, and response to intestinal injury were assessed, with attention to sex-specific differences. Effects of cholinergic and muscarinic agonism on epithelial cell homeostasis were evaluated employing murine and human colonoids.

**Results:**

Genetic epithelial ablation of the M3R employing Vil-Cre × M3R fl/fl mice interestingly resulted in the prominent reduction in *Lgr5*-expressing progenitor cells in male tissues, contrasting an expansion of *Lgr5*-expressing cells in female colonic epithelia. This difference was abrogated in young female Vil-Cre × M3R fl/fl mice, which harbor reduced circulating sex hormone levels. Genetic M3R ablation further induced changes to epithelial differentiation. Importantly, male Vil-Cre × M3R fl/fl mice developed severe inflammation following induction of acute experimental colitis, which did almost not affect female Vil-Cre × M3R fl/fl mice. Moreover, sex-specific effects of modulations of cholinergic and muscarinic signaling on epithelial cells could be corroborated in murine and human colonoids.

**Conclusions:**

Our data reveal sex differences in the modulation of intestinal, colonic epithelial cells by cholinergic, muscarinic signaling and highlight the potential for therapeutic strategies targeting cholinergic receptor signaling in colonic inflammatory diseases.

## Graphical Abstract

**Graphical Abstract:**
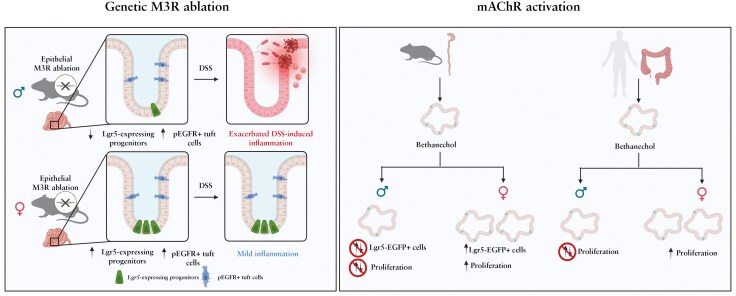

## 1. Introduction

Intestinal stem cells (ISCs) and progenitor cell types, located at the intestinal crypt base, are exposed to diverse signals that contribute to their proliferation and differentiation.^1^ These signals originate from the epithelial and non-epithelial stem cell niche, which comprised enteric neurons, myofibroblasts, or endothelial cells.^2,3^

Acetylcholine (ACh) is a major neurotransmitter in the enteric nervous system (ENS)^4^ that plays an important role in the gut through the activation of muscarinic (mAChRs) and nicotinic receptors (nAChRs)^5^ on target cells. Mammalian mAChRs consist of 5 main subtypes (M1R–M5R), which are coupled to G-proteins.^6^ mAChRs are not exclusively localized in cell types of the ENS but also in intestinal epithelial cells (IECs), supporting a major involvement in intestinal homeostasis and barrier maintenance.^7,8^ M3R is one of the major mAChRs that are expressed in IECs,^9–11^ and there is growing evidence of its importance in regulating intestinal mucosal homeostasis.^9,12–14^ For instance, we could recently observe that genetic, epithelial ablation of the M3R induced prominent changes to small intestinal epithelial differentiation with the expansion of DCLK1+ tuft cells, which appeared to represent a compensatory mechanism to maintain the cholinergic stem cell niche.^9^ In line, modulations of muscarinic receptor signaling have been reported to impact intestinal regeneration. As such, accumulation of proinflammatory cytokines and delayed parasite clearance was observed in the small intestine of whole-body M3R knockout mice infected with *Nippostrongylus brasiliensis.*^13^ Similarly, drug-induced inhibition of mAChRs aggravated susceptibility to infection with *Staphylococcus aureus.*^15^ Moreover, whole-body genetic ablation of M3R resulted in exacerbated DSS-induced colitis.^12^ Takahashi et al. examined the effect of whole-body M3R ablation on small intestinal IECs and reported a potential mechanistic basis for its importance in maintaining intestinal barrier integrity by interacting with the EphB/ephrin-B signaling pathway.^14^ Beside M3R, M1R is also reported to play a critical role in intestinal homeostasis.^16,17^ While these studies highlight the modulation of intestinal homeostasis and regeneration by cholinergic, muscarinic receptor signaling, potential sex-specific and regional differences in the muscarinic modulation of IEC homeostasis and regeneration remain largely unknown.

Several studies highlight sexual dimorphism in the gut which might explain sex-specific variations in intestinal disorders. ISCs of female *Drosophila* and female mice exhibited higher proliferative capacity than in males.^18,19^ Furthermore, estrogen is implicated in the amelioration of dextran sodium sulfate (DSS) induced colitis in ovariectomized mice.^20^ Sexual dimorphism has been observed in the functions of mAChRs in the brain. While female rats exhibited an increased number of muscarinic receptor binding sites in response to chronic ethanol intake, male rats showed no difference.^21^ Further, treating rats with scopolamine, a cholinergic muscarinic receptor antagonist, induced behavioral changes in males but not in females.^22^ In addition to the influence of biological sex, region-specific differences in intestinal segment physiology are reported to impact ISC dynamics and the susceptibility to intestinal disorders.^23–25^ Azkanaz et al. observed regional variations in ISC functionality between the small and large intestine due to retrograde movement.^23^ Moreover, small intestinal ISCs display higher radiosensitivity than colonic ISCs.^26^ These observations suggest that both sex and regional factors influence ISC behavior, yet how modulations by niche factors such as cholinergic signaling may exert sex-specific or regionally different effects on ISC and progenitor cells remains poorly understood. Therefore, we here investigated how cholinergic, muscarinic signaling affects colonic IEC homeostasis, which indeed revealed regional differences as compared to previous data on small intestinal tissues.^9^ More importantly, genetic epithelial ablation of the M3R induced sex-specific changes to colonic IEC homeostasis and regeneration in vivo, which translated in more prominent modulations of female murine and human colonoids in vitro by cholinergic agonism. Modulations of cholinergic, muscarinic signaling thus appear promising to foster intestinal regeneration, yet highlight the importance to take regional as well as sex-specific effects of IEC modulations by niche factors into account.

## 2. Materials and methods

### 2.1. Animal experiments

C57BL/6J (Strain #000664), Lgr5-EGFP-IRES-CreERT2 (Strain #008875), Prox1-CreERT2 (Strain #022075), and R26-tdTom (Strain #007909) mice were purchased from the Jackson Laboratory (USA). Vil-Cre × M3R × M1R fl/fl mice were obtained under an MTA from Columbia University (Prof. Timothy Wang, USA), rederived and employed to derive Vil-Cre × M3R fl/fl as well as Lgr5-EGFP-IRES-CreERT2 x M3R fl/fl and Prox1-CreERT2 x M3R fl/fl strains, respectively. All mice were maintained under specific pathogen-free conditions at a regular 12-hour light–dark cycle with access to food and water ad libitum. All animal experiments were approved by the District Government of Upper Bavaria (Vet 02-19-117) and performed in compliance with the ARRIVE guidelines and the German Animal Welfare and Ethical Guidelines of the Klinikum rechts der Isar, TUM, Munich, Germany.

Acute experimental colitis was induced in wild-type C57BL/6J and Vil-Cre × M3R fl/fl mice by administration of 1% (w/v) dextran sodium sulfate (DSS, MP Biomedicals) in drinking water for 7 days. On day 8, DSS water was replaced with normal drinking water for 5 days. Body weight was measured daily, and stool consistency and fecal occult blood were assessed using the Guaiac test (CARE diagnostica). Animals that lost more than 20% of their initial weight were euthanized.

Conditional M3R deletion was induced by a single oral administration of tamoxifen (3 mg, Sigma-Aldrich) dissolved in corn oil. Animals were sacrificed after 5 days of tamoxifen induction.

### 2.2. Intestinal crypt isolation

Harvested tissue fragments were finely chopped, washed in ice-cold DPBS, and treated with EDTA (Invitrogen) chelation solution (30 mM) with gentle agitation at 4 °C. Subsequently, the suspensions were filtered (100 μm, Greiner Bio-One), centrifuged, and then resuspended in RLT (Qiagen) for RNA extraction or RIPA for Western Blot analysis.

### 2.3. Murine intestinal organoid culture

Intestinal crypts were generated from the proximal colon of age and sex-matched Lgr5-EGFP-IRES-CreERT2 and wild-type C57BL6/J mice. In short, colonic fragments were washed several times in cold 1 × PBS and then incubated in 25 mL of EDTA PBS (30 mM) at 4 °C for 30 min. Intestinal fragments were resuspended in 10% (V/V) FBS in PBS and passed through 100 µm filters. Crypt suspensions were pelleted and resuspended in Corning Matrigel Growth Factor Reduced Basement Membrane Matrix. Droplets of 50 µL were plated on 24-well plates (Corning), overlayed with L-WRN medium, and incubated at 37 °C, 5% CO_2_.

Colonic organoids were maintained on L-WRN medium overnight. Consequently, the medium was replenished daily with fresh Bch-containing medium (250 nM) for 4 days. Supernatants were collected and organoids were harvested for RNA isolation and flow cytometry analysis.

### 2.4. Human intestinal organoid culture

Tissue biopsies from the colon were collected by endoscopy from healthy controls from the Department of Internal Medicine II, TUM University Hospital, Klinikum rechts der Isar, TUM School of Medicine and Health, Technical University of Munich. Samples were collected upon receiving informed consent from each patient. Protocols were approved by the ethics committee of TUM University Hospital, Klinikum rechts der Isar (2021-93_1-S-NP; 2018-322_8-S-SB). All experiments were performed in accordance with relevant guidelines and regulations.

Minced tissue was digested in 2 mM EDTA in DPBS on ice for 20 minutes. Next, the suspension was flushed with cold 10% (v/v) FBS in PBS and was filtered using a 100-µm cell strainer. Crypt suspensions were pelleted and resuspended in the Corning Matrigel Growth Factor Reduced Basement Membrane Matrix. Droplets of 50 µL were plated on 24-well plates (Corning), overlayed with L-WRN medium, and incubated at 37 °C, 5% CO_2_ for 7 days with medium exchange every 2–3 days.

For Bch treatment experiments, established organoid lines (passage 3 or more) were passaged and incubated with a standard L-WRN medium overnight. The medium was exchanged with fresh medium supplemented with Bethanechol (250 nM). The medium was replenished daily with fresh Bch-containing medium for 4 days.

### 2.5. Flow cytometry of colonoids and cell sorting

To generate a single-cell suspension, organoids were harvested and incubated with cold Gentle Cell Dissociation Reagent (GCDR, STEMCELL Technologies) on ice for 10 minutes. Then, organoids were pelleted by centrifuging for 5 minutes at 200 × g at 4 °C. The pellet was resuspended in 5 mL of cold DMEM/F12 + 15 mM HEPES. Samples were centrifuged for 5 minutes at 200 × g at 4 °C. The cell suspension was incubated with TrypLE (Thermo Fisher Scientific) at 37 °C for 5 minutes. Samples were briefly vortexed and DMEM/F12 + 10% (v/v) FBS was added and vigorously mixed. Subsequently, cells were pelleted and incubated with live/dead fixable violet dead cell stain kit (1:1,500, Thermo Fisher Scientific) on ice in the dark for 20 minutes. Events were measured using BD FACSCanto™ II flow cytometer (BD Biosciences) and analyzed by FlowJo software. FACS was performed using BD FACSAria Fusion (BD Bioscience).

The proliferation rate was measured using Click-iT™ Plus EdU Pacific Blue kit (C10636, Thermo Fisher Scientific) according to the manufacturer’s instructions.

### 2.6. L-lactate assay

The L-lactate level was measured in organoid culture supernatants (1:10 v/v) using the kit from Megazyme (K-LATE) according to the manufacturer’s instructions.

### 2.7. Histology and microscopy analyses

Immunohistochemical staining was performed utilizing paraformaldehyde-fixed and paraffin-embedded tissue sections (5-10 µm). Slides were deparaffinized and dehydrated in decreasing concentrations of ethanol according to standard protocols. To retrieve antigens, the slides were subjected to boiling in citrate buffer (10 mM, pH 6.0; Vector laboratories) using a microwave for 5 minutes at 900W followed by 12 minutes at 360W. Endogenous peroxidase was blocked by incubation with 3% (v/v) hydrogen peroxide (Sigma-Aldrich) for 10 minutes. Sections were blocked for 1 hour with 3% (v/v) BSA (Fisher Scientific) in 0.1% (v/v) TritonX-100 in PBS (PBST). Primary antibodies (Table 1) were diluted in 3% BSA in PBST and incubated overnight at 4 °C. The next day biotinylated secondary antibodies (Table 1) were incubated for 30 minutes at RT. Subsequently, the slides underwent incubation with the ABC kit (Vector Laboratories) and were then visualized using 3,3′-diaminobenzidine (DAB, Sigma-Aldrich) as the chromogen. Slides were counterstained with hematoxylin, rehydrated in increasing ethanol, and mounted for viewing. Human colonic tissue sections were provided by the Institute of Pathology of the Technical University Munich and the analysis was approved by the ethics committee of Klinikum rechts der Isar (2021-93_1-S-NP).

**Table 1.** List of antibodies.

Primary antibodies	Host species	Company	Working concentration
α-Tubulin (11H10)	Rabbit	Cell Signaling	1:1000 (WB)
DCAMKL1 (C-Term)	Rabbit	Abcepta	1:500 (IHC)
M3R	Rabbit	Abcam	1:200 (IHC, IF)
P-EGFR (Tyr845)	Rabbit	Thermofisher	1:200 (IHC, IF)
PI3 Kinase p85 beta	Rabbit	Abcam	1:1000 (WB)
**Secondary antibodies/** **labeled polymers**		**Company**	**Working concentration**
Anti-rabbit IgG HRP		Cytiva	1:5000 (WB)
Alexa Fluor 594 goat anti-rabbit IgG		Invitrogen	1:500 (IF)

*Lgr5* ISH was performed using the RNAscope 2.5 LS Reagent Kit–BROWN (322100, Advanced Cell Diagnostics) on an automated Leica BOND RX^m^ system according to the manufacturer’s instructions at the Institute of Pathology, TUM School of Medicine and Health, Technical University of Munich. After deparaffinization and dehydration, colonic sections were incubated with the Lgr5 probe (312178, Advanced Cell Diagnostics) for 2 hours at 42 °C. Next, the signal was amplified for 30 minutes at 42 °C followed by detection using DAB staining. Slides were counterstained with hematoxylin.

Bright-field images were acquired using a microscope (Imager A1; Zeiss) connected to a camera (AxioCam; Zeiss) using AxioVision software.

For immunofluorescence, OCT-embedded intestinal sections were fixed and deparaffinized as described above. Washed slides were permeabilized and blocked simultaneously for 1 hour with 3% (v/v) BSA (Fisher Scientific) in PBST. Primary antibodies (Table 1) were applied for overnight staining at 4 °C in 3% BSA in PBST. The next day, fluorophore-conjugated secondary antibody (Table 1) at a concentration of 1:500 was added for 1h at RT. Slides were counterstained and mounted with Vectashield anti-fade DAPI-containing mounting medium (Vector Laboratories).

H&E-stained colonic tissue sections from experimental mice following induction of colitis were blindly scored as previously published^27^ with the evaluation of the following criteria: crypt architecture (0 for normal to 3 for severe distortion with crypt loss), mucosal inflammatory cell infiltration (0 for normal to 3 for dense infiltrate), submucosal inflammatory cell infiltration (0 for normal to 2 for dense infiltrate) muscle thickening (0 if crypt base rests on muscularis mucosae to 1 for marked thickening), epithelial damage (0 for absent to 3 for ulcerations). The total histological damage score is the sum of all individual scores.

### 2.8. Acetylcholine ELISA

The acetylcholine level was measured in the colon using Ach ELISA (E4453-100, Biovision) according to the manufacturer’s instructions. Colonic tissue was homogenized in 300µl PBS. Samples were diluted 1:5 in dilution buffer and conjugated with HRP-conjugate. After 60 minutes of incubation at 37 °C, wells were washed and absorbance was measured at 450 nm after applying chromogen solution. Protein concentration was determined using a provided standard curve. Values were normalized to sample weights.

### 2.9. KC ELISA

KC levels were measured in the serum of DSS-treated mice (1:5) using mouse CXCL1/KC Quantikine ELISA Kit (MKC00B, R&D Systems) according to the manufacturer’s instruction.

### 2.10. Western blot

Protein was extracted from colonic epithelial-enriched isolates by resuspension in RIPA Buffer supplemented with phosphatase and protease inhibitors (Sigma-Aldrich). Protein concentration was determined by Pierce™ BCA kit (Thermo Fisher Scientific). A mixture of protein samples (50 µg) and Laemmli buffer (Sigma-Aldrich) was loaded onto a precast polyacrylamide gel. Proteins were separated by gel electrophoresis and blotted to polyvinylidene difluoride (PVDF) membranes according to standard protocols. After blotting, membranes were blocked in 5% (w/v) non-fat milk or 5% (w/v) BSA in Tween-TBS (T-TBS) for 1 hour at RT. Diluted primary antibodies in 5% non-fat milk or 5% BSA in T-TBS were applied to membranes and incubated overnight at 4 °C (Table 1). Following incubation, membranes were washed with T-TBS and incubated with respective horse-radish peroxidase (HRP)-conjugated secondary antibodies (Table 1) for 1 hour at RT. Subsequently, blotted membranes were washed with T-TBS. HRP substrate solution was evenly distributed on membranes and chemiluminescence was detected in the ChemiDocTM XRS + imaging system using the ImageLab™ software. Intensity of chemiluminescence was quantified via t ImageJ software and expression levels of proteins of interest were normalized to housekeeper proteins.

### 2.11. PI3K/AKT array

Phosphorylated proteins involved in the PI3K/AKT pathway were assessed by applying protein isolates to the Human/Mouse AKT pathway phosphorylation array C1 (AAH-AKT-1-2, RayBiotech). Protein isolates from colonic crypts of WT and Vil.M3R were processed according to the manufacturer’s instructions. The signal was detected using ChemiDocTM XRS + imaging system.

### 2.12. RNA extraction, cDNA synthesis, and qPCR

RNA isolation was performed using RNeasy Mini kit and RNeasy Micro kit (Qiagen) according to the manufacturer’s protocol. RNA concentrations were measured using NanoDrop 2000 (Thermo Fisher Scientific). cDNA was synthesized with QuantiTect Rev. Transcription kit (Qiagen) using 200-500 ng RNA. Target genes were amplified on qTOWER3 G real-time thermal cycler (Analytik Jena) by utilizing QuantiFast SYBR Green RT-PCR kit (Qiagen) and respective forward and reverse primers (Table 2). Ct-Values of targets were normalized to *RPLP0* or *Hmbs* (Table 2) that served as an internal housekeeping gene via the 2 − dCT. Primers were purchased from Sigma-Aldrich.

**Table 2.** List of oligonucleotides.

Target gene (Human)	Forward primer (5′-3′)	Reverse primer (5′-3′)
*RPLP0*	GCGTCCTCGTGGAAGTGACA	GCGACCTGGAAGTCCAACTA
*CHRM1*	GCCATCCTCCTGGCCTTCAT	GGCTTTGTTGCAGAGTGCGT
*CHRM3*	ACAGGAGGAAGTATGGCCGC	ACTGCTGCTGTGGTCTTGGT
**Target gene (mouse)**	**Forward primer (5′-3′)**	**Reverse primer (5′-3′)**
*Ascl2*	AGTACATTCGGACCCTCTCTC	CGTACCAGTCAAGGTGTGCTT
*Hmbs*	TTGGAAAGACCCTGGAAACC	TGAATTCCTGCAGCTCATCC

### 2.13. RNA sequencing analysis

RNA sequencing was conducted following previously established protocols.^28,29^ In summary, cDNA was synthesized using oligo-dT primers with sample barcodes, unique molecular identifiers (UMIs), and adaptors. The cDNA was subsequently amplified and fragmented using the NEB UltraTM II FS kit. Sequencing was performed on a NextSeq 500 (Illumina) with 65 cycles for read1 (cDNA) and 19 cycles for read2 (barcodes and UMIs). Drop-seq pipeline (v1.0) was used to process the data and generate UMI tables.^30^ Alignment was performed against the reference genome GRCm38 and GRCh38, with gene annotations based on GENCODE M25 and Human Release 38, respectively. Genome-wide differential gene expression analysis was calculated using the DESeq2 R package^31^ for RNA Seq count data, considering a false discovery rate (FDR) of ≤ 0.05 for significance. Designs accounted for identifier and treatment, respectively. PCA was carried out using the plotPCA function from DESeq2. Genome-wide differential gene expression signatures as represented by Wald statistics per gene were interrogated by gene set enrichment analysis^32,33^ using modules “c2.cp”, “c3.tft.gtrd”, “c6.al”, and “h.all” from the MSigDb version 7.4.^34^

### 2.14. Graphical abstracts

All schematics were created with BioRender.com.

### 2.15. Statistical analysis

Statistical analysis was performed with GraphPad Prism version 9 (San Diego, CA, USA). Statistical differences of normally distributed data were assessed by *t*-test while Mann–Whitney *U* test was used for not-normally distributed data. The specific statistical test employed for each graph is outlined in the respective figure legend. Differences between groups were considered to be significant at a *P*-value of * < .05, ** <.01, *** <.001, or **** <.0001.

## 3. Results

### 3.1. Epithelial genetic M3R ablation induces sex-specific modulation of colonic *Lgr5*-expressing progenitor cells

**Figure 1.**
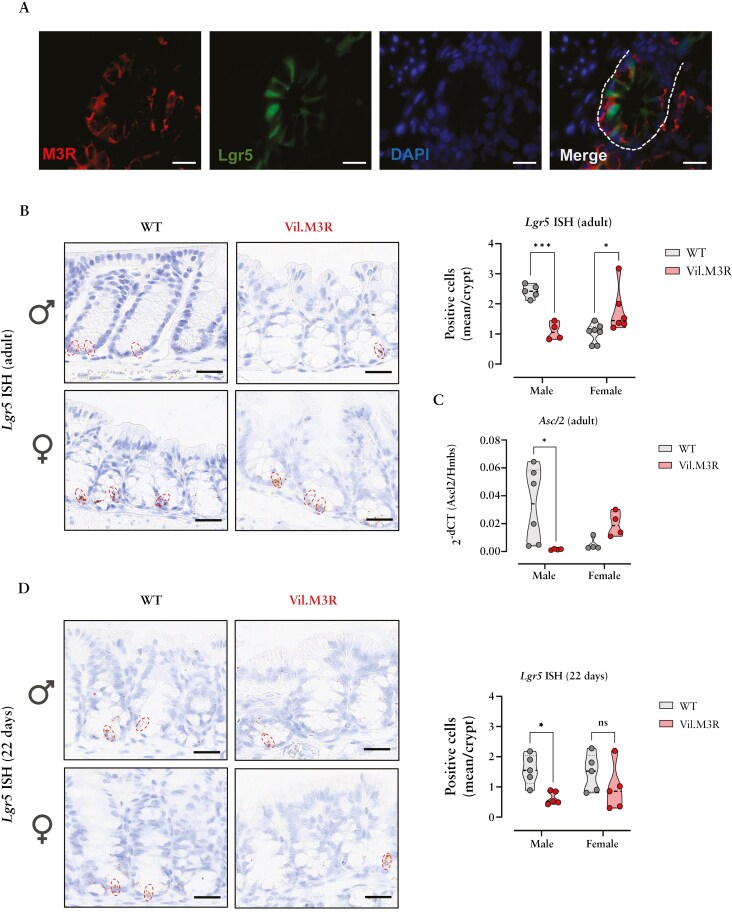
**Sex-specific regulation of colonic Lgr5 + progenitor cells by epithelial M3R ablation.** (**a**) Colonic section from Lgr5-EGFP-IRES-CreERT2 mice was stained with M3R (red). Nuclei were marked with DAPI (blue). Dashed line represents a colonic crypt. Scale bar = 20 µm (40×). (**b**) Representative images of Lgr5 ISH staining of colon harvested from adult WT and Vil.M3R mice. Scale bar = 20 µm (40×). *Lgr5*-expressing progenitor cells (red circles) were counted at the base of the colonic crypts. (**c**) qPCR was performed to measure mRNA levels of *Ascl2* in colonic epithelial extracts from adult WT and Vil.M3R mice. (**d**) Colonic levels of *Lgr5*-expressing progenitor cells (red circles) were measured in young WT and Vil.M3R mice (22 days). Scale bar = 20 µm (40×). Statistical differences were detected by 2way ANOVA with Šídák’s multiple comparisons test. All results are shown as truncated violin plots with median represented as dashed line. *P < .05, ***P < .001.

ISCs and progenitor cells are known to express *Chrm3* (gene encoding M3R), which plays a key role in supporting their self-renewal.^5,9,11^ The role of mAChRs, and M3R in particular, was considerably investigated previously in small intestinal homeostasis.^9,14^ However, little is known about the contribution of M3R signaling to colonic homeostasis. First, we confirmed positive co-labeling of M3R with Lgr5-EGFP + progenitor cells in proximal colonic tissue slides from adult Lgr5-EGFP-IRES-CreERT2 mice (Figure 1a). Next, we employed Vil-Cre × M3R fl/fl mice (Vil.M3R) to investigate the effect of IEC-specific genetic ablation of M3R on colonic ISCs and progenitor cells. Interestingly, epithelial deletion of M3R in vivo induced sex-specific differences in colonic *Lgr5*-expressing progenitor cell numbers. While male Vil.M3R mice exhibited a severe reduction in colonic *Lgr5*-expressing progenitor cells, female mice showed the opposite phenotype with an expansion of *Lgr5*-expressing cells (Figure 1b). Moreover, mRNA expression of *Ascl2*, another well-established stem cell marker,^35^ showed distinct expression levels in isolated crypts from Vil.M3R mice from both genders similar to *Lgr5* gene expression (Figure 1c). In view of the reported influence of sex hormones on ISC regulation,^36,37^ we next aimed to dissect the effect of sex hormones in Vil.M3R mice and assessed the impact of genetic M3R ablation in young Vil.M3R mice (22 days of age) that displayed low levels of circulating testosterone and estradiol as previously described^38^ (Figure S1a,b). Notably, male pups still exhibited a significant reduction in colonic *Lgr5*-expressing cells, while females showed a moderate decrease in *Lgr5*-expressing cells as opposed to adult tissue samples (Figure 1d).

### 3.2. Colonic epithelial M3R ablation induces compensatory pEGFR + tuft cell expansion and Ach production

**Figure 2.**
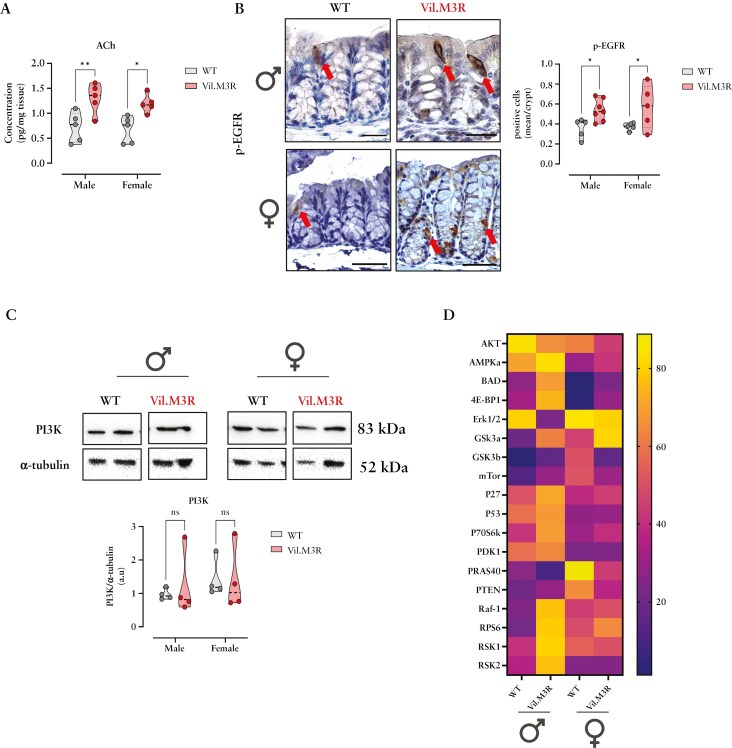
**Colonic epithelial M3R ablation leads to tuft cell expansion and increased acetylcholine production.** (**a**) ACh levels were measured by ELISA in colonic tissue harvested from WT and Vil.M3R mice. (**b**) Representative images of p-EGFR immunostaining of colon harvested from WT and Vil.M3R mice with red arrows showing p-EGFR + cells. Scale bar = 50 µm (40×). p-EGFR positive tuft cells were counted along colonic crypts. (**c**) Western blot was performed from colonic protein extracts from WT and Vil.M3R animals with respective antibodies. Relative protein expression of PI3K was calculated compared to α-Tubulin. (**d**) Heatmap of the average abundance of phosphorylated proteins involved in PI3K/Akt pathway in colonic protein extracts from male and female WT and Vil.M3R mice. Statistical differences were detected by 2way ANOVA with Šídák’s multiple comparisons test. Results are shown as truncated violin plots with median represented as dashed line. *P < .05, **P < .01.

Since we observed distinct effects of genetic M3R ablation on colonic progenitor cell types in a sex-dependent manner, we next sought to examine its effect on the cholinergic niche and epithelial differentiation. Similar to the small intestine,^9^ epithelial ablation of M3R led to a significant upregulation of colonic ACh synthesis (Figure 2a). Tuft cells have been identified to be equipped to produce ACh and have been proposed to regulate intestinal homeostasis via muscarinic receptors.^39^ In line with potential regional differences in intestinal tuft cells,^40^ we could observe the expansion of phospho-EGFR (p-EGFR) positive tuft cells in colonic tissues of male and female Vil.M3R mice (Figure 2b). On the other hand, immunostaining for DCLK1, another prominent tuft cell marker, revealed the opposite trend, whereby the number of colonic DCLK1 + cells decreased in male Vil.M3R and appeared unchanged in females compared to controls (Figure S2). These observations contrasted our previous findings from small intestinal tissues of similar transgenic mice.^9^

Next, we aimed to identify progenitor cells that potentially sensed genetic cholinergic perturbation and orchestrated the observed p-EGFR + tuft cell expansion. Given the observed variations in *Lgr5*-expressing progenitor cells resulting from epithelial genetic M3R ablation, we first examined Lgr5-EGFP + progenitor cells as potential sensors of cholinergic perturbation. Indeed, conditional M3R deletion in Lgr5-EGFP + progenitor cells (Lgr5-EGFP-IRES-CreERT2 x M3R fl/fl mice) resulted in a moderate expansion of colonic p-EGFR + tuft cells after 5 days of tamoxifen induction (Figure S3a). In contrast, conditional M3R deletion in Prox1-positive progenitor cells (Prox1-CreERT2 x M3R fl/fl mice) did not result in an expansion of colonic p-EGFR + tuft cells (Figure S3b). These data pointed to colonic Lgr5-EGFP + progenitor cells sensing genetic cholinergic receptor ablation, which prominently contrasts the sensing of Prox1-positive progenitors in the small intestine as observed previously.^9^

The PI3K/Akt pathway is a major pathway involved in stem cell maintenance and expansion.^41,42^ Moreover, the PI3K cascade is closely linked to tuft cell differentiation.^9,43^ Hence, we speculated that the changes observed in tuft cells due to altered M3R signaling may relate to downstream activation of the PI3K signaling axis, similar to our observations in small intestine.^9^ Nonetheless, in contrast to small intestine, colonic PI3K abundance appeared comparable between WT and Vil.M3R in both genders (Figure 2c). Moreover, we ran an in-depth analysis using a phosphoprotein array to investigate proteins involved in the PI3K/Akt pathway. This analysis specifically measured phosphorylation states rather than total protein levels, highlighting functional changes in signaling pathways. Interestingly, we could detect pathway changes which appeared, however, more prominent in colonic lysates of male Vil.M3R mice, including the upregulation of phosphorylation levels of BAD, 4E-BP1, Raf-1, GSK3α, RP S6, or RSK1/2 (Figure 2d). Phosphoproteins such as ERK1/2 showed downregulated in male mice with epithelial M3R deletion (Figure 2d). On the other hand, female Vil.M3R tissues exhibited absent or milder pathway changes, especially in regard to the phosphorylation of ERK1/2, BAD or 4E-BP1 (Figure 2d). Collectively, this data points to potential regional differences in sensing cholinergic signaling interruptions by intestinal epithelial progenitor cell types, which correlates with regionally distinct tuft cell expansion and potential sex-specific changes to intracellular PI3K/Akt signaling.

### 3.3. Genetic M3R ablation does not sensitize female mice to acute intestinal injury

**Figure 3.**
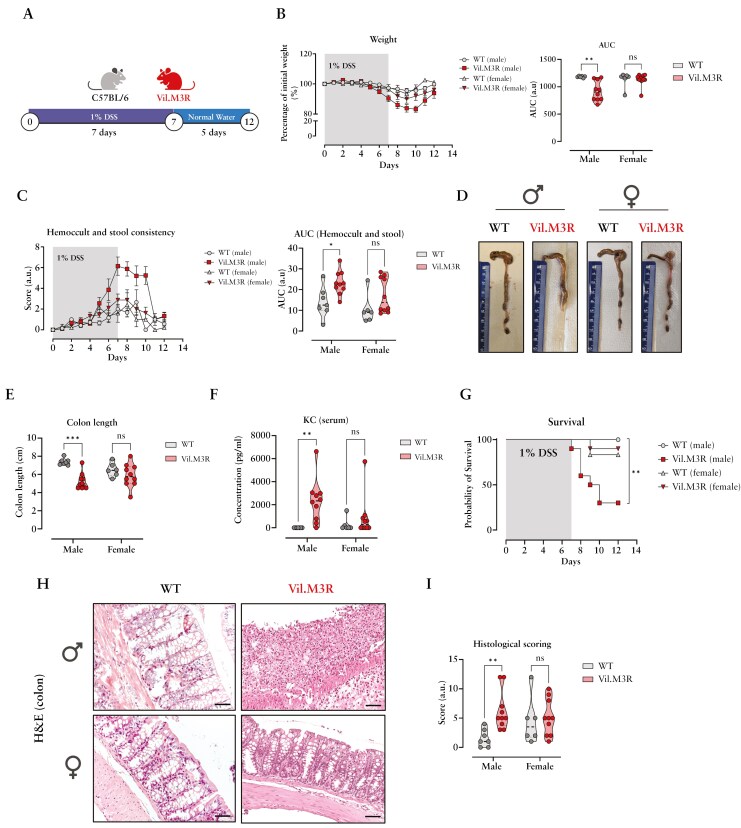
**Acute DSS-induced colitis induces sex-specific phenotypes in mice with epithelial M3R ablation.** (**a**) Acute experimental colitis was induced in WT and Vil.M3R mice from both sexes by dissolving 1% DSS in the drinking water for 7 days followed by 5 days of normal drinking water. (**b**) Body weights were monitored daily and reported as a percentage of the initial body weight before adding DSS. The area under the curve (AUC) was determined for each animal. (**c**) Stool consistency and the presence of blood in stool were evaluated throughout the experiment. AUC was measured for each animal. (**d**) Representative images of colon from DSS-treated mice. (**e**) Colon lengths were assessed in WT and Vil.M3R on day 12. (**f**) ELISA was performed to measure serum levels of KC in DSS-treated animals. (**g**) Survival rates of DSS-treated animals were evaluated. Mice that lost ≥ 20% of their initial body weight were euthanized. (**h**) H&E staining of colonic sections from WT and Vil.M3R animals and (**i**) colon histopathological scoring was conducted. Scale bar = 50 µm (20x). Data are shown as either mean ± SEM or truncated violin plots with median represented as dashed line. Statistical differences were detected by 2way ANOVA with Šídák’s multiple comparisons test except (**g**) Log-rank (Mantel-Cox) test. *P < .05, **P < .01, ***P < .001.

Lgr5-EGFP + progenitor cells have been identified to play a critical role in the repair of the intestinal epithelium following intestinal injury.^44^ Several studies highlighted the importance of muscarinic receptors for the maintenance of intestinal mucosal barrier integrity and epithelial regeneration.^12,16,45,46^ Therefore, we next employed an acute colitis model to assess whether the observed differences in *Lgr5*-expressing cell numbers as well as PI3K/Akt pathway members between male and female Vil.M3R would differentially affect intestinal inflammation severity. The acute colonic injury was induced by treating male and female WT and Vil.M3R mice with 1% DSS in drinking water for 7 days, followed by 5 days of normal drinking water (Figure 3a). Interestingly, male Vil.M3R mice exhibited strongly exacerbated manifestations of intestinal inflammation, namely weight loss, rectal bleeding, and diarrhea compared to male controls (Figure 3b,c). A significant weight loss was observed starting from day 6 and reaching its highest peak of 17% on day 10 (Figure 3b). Similarly, male epithelial M3R-ablated animals showed severe rectal bleeding spanning from day 7 to day 10 (Figure 3c). Conversely, female Vil.M3R mice were only mildly affected by DSS compared to their control counterparts (Figure 3b,c). Furthermore, another indication of inflammation severity is colon length shortening.^47^ While male Vil.M3R showed severe colon length shrinkage, female animals were comparable between groups (Figure 3d,e). Acute DSS colitis displays an upregulated Th1-Th17 response with elevation of several proinflammatory cytokines, including Keratinocyte-derived chemokine (KC).^48^ Accordingly, accumulation of the proinflammatory cytokine KC was prominent in the serum of treated male Vil.M3R mice indicating a severe systemic inflammation (Figure 3f). Around 70% of the male mice with epithelial ablation of M3R were euthanized due to severe weight loss which exceeded 20% of the initial body weight (Figure 3g). On the other hand, 90% of female Vil.M3R mice survived to the last day of the experiment (Figure 3g). Histopathological evaluation of H&E-stained colonic sections from DSS-treated animals revealed similar findings with massive mucosal accumulation of immune cells and distortion of the crypt architecture in male Vil.M3R mice (Figure 3h,i).

### 3.4. Lgr5-EGFP + progenitor cell expansion and transcriptional activation is prominent in female colonic organoids upon muscarinic activation

**Figure 4.**
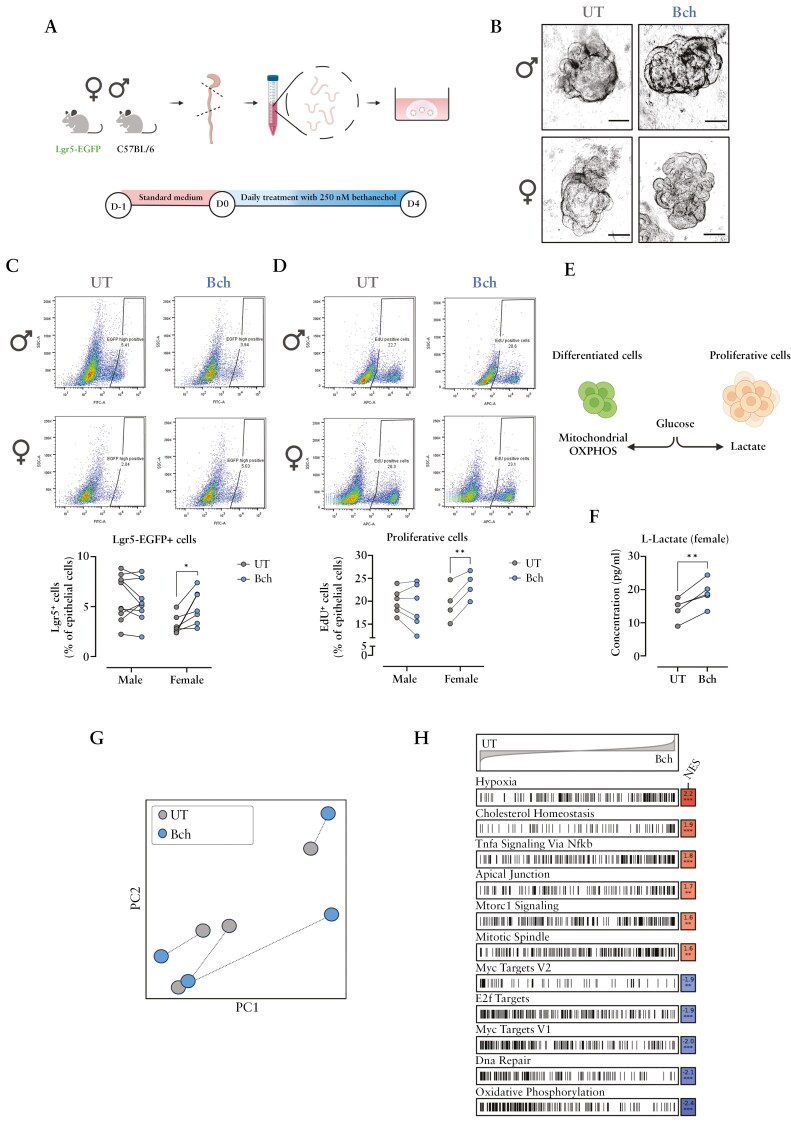
**Muscarinic activation increased Lgr5-EGFP + cells and proliferation in female-derived colonic organoids.** (**a**) Colonic organoids were generated from the proximal colon of Lgr5-EGFP-IRES-CreERT2 and WT C57BL6/J mice and incubated with standard growth medium for 1 day. Subsequently, they were treated daily with Bch (250 nM) for 4 days. (**b**) Representative microscopic images of untreated (UT) vs Bch-treated colonoids on day 4. Scale bar = 100 µm (20×). (**c**) Lgr5-EGFP + progenitor cells were detected by flow cytometry in single-cell suspension from male and female colonoids. (**d**) Proliferative capacity was measured by EdU assay. (**e**) Summary of glucose metabolism in proliferative and differentiated cells. (**f**) Lactate levels were measured in the supernatants of UT and Bch-treated organoids. (**g**) PCA of transformed count data from bulk RNA sequencing of Lgr5-EGFP + progenitor cells isolated from UT and Bch-treated female colonoids. (**h**) Hallmark gene-set enrichment analysis of differential gene expression in Lgr5-EGFP + progenitor cells isolated from untreated (UT) and Bch-treated female colonoids. Statistical differences were detected by multiple paired *t* tests. *P < .05, **P < .01. UT: untreated; Bch: bethanechol; NES = normalized enrichment score.

Muscarinic activation was reported to upregulate ISC proliferation.^49,50^ In view of our in vivo data, we hypothesized that the observed differences in *Lgr5*-expressing cells between genders may either originate from direct cellular effects of genetic M3R ablation (Figure 2) or differences in the effect of cholinergic stimulation of remaining cholinergic receptors by increased Ach tissue levels, in addition to circulating sex hormones. Therefore, we next employed the intestinal organoid system^51^ to specifically test the effect of cholinergic, muscarinic receptor activation on Lgr5-EGFP + cells and epithelial proliferation. Murine colonic organoids generated from proximal colon were treated with 250 nM bethanechol (Bch), a non-selective mAChRs agonist, for 4 days (Figure 4a). Morphologically, Bch-treated male-derived organoids appeared larger in size than untreated controls (Figure 4b). Interestingly, treatment with Bch resulted in an expansion of Lgr5-EGFP + cells and increased proliferation in female-derived colonoids, whereas no such effect was observed in organoids from male mice (Figure 4c,d). Metabolic changes are known to drive IEC proliferation.^52^ ISC proliferation is associated with upregulated glycolysis, while differentiated IEC display boosted mitochondrial respiration.^53,54^ Interestingly, metabolic analysis revealed a significant increase in the glycolysis byproduct L-lactate concentration in the supernatant of female colonic organoids treated with Bch (Figure 4e, f). Next, we performed bulk RNA sequencing analysis of sorted Lgr5-EGFP + cells from non-treated (UT) and Bch-treated colonoids. Indeed, principal component analysis (PCA) showed a distinct genetic profile of Bch-treated female organoids (Figure 4g). Bch-treated female Lgr5-EGFP + cells exhibited enhanced activity in pathways associated with hypoxia, cholesterol homeostasis, TNF-alpha/NF-κB, apical junction, mTORC1, and mitotic spindle (Figure 4h). On the other hand, pathways such as Myc, E2F, DNA repair or oxidative phosphorylation were downregulated in Lgr5-EGFP + cells treated with Bch (Figure 4h). In contrast, RNA sequencing analysis did not reveal prominent changes in Bch-treated colonic, male Lgr5-EGFP + cells (see deposited data).

### 3.5. Treatment with bethanechol enhances the proliferative capacity of human female-derived colonoids

**Figure 5.**
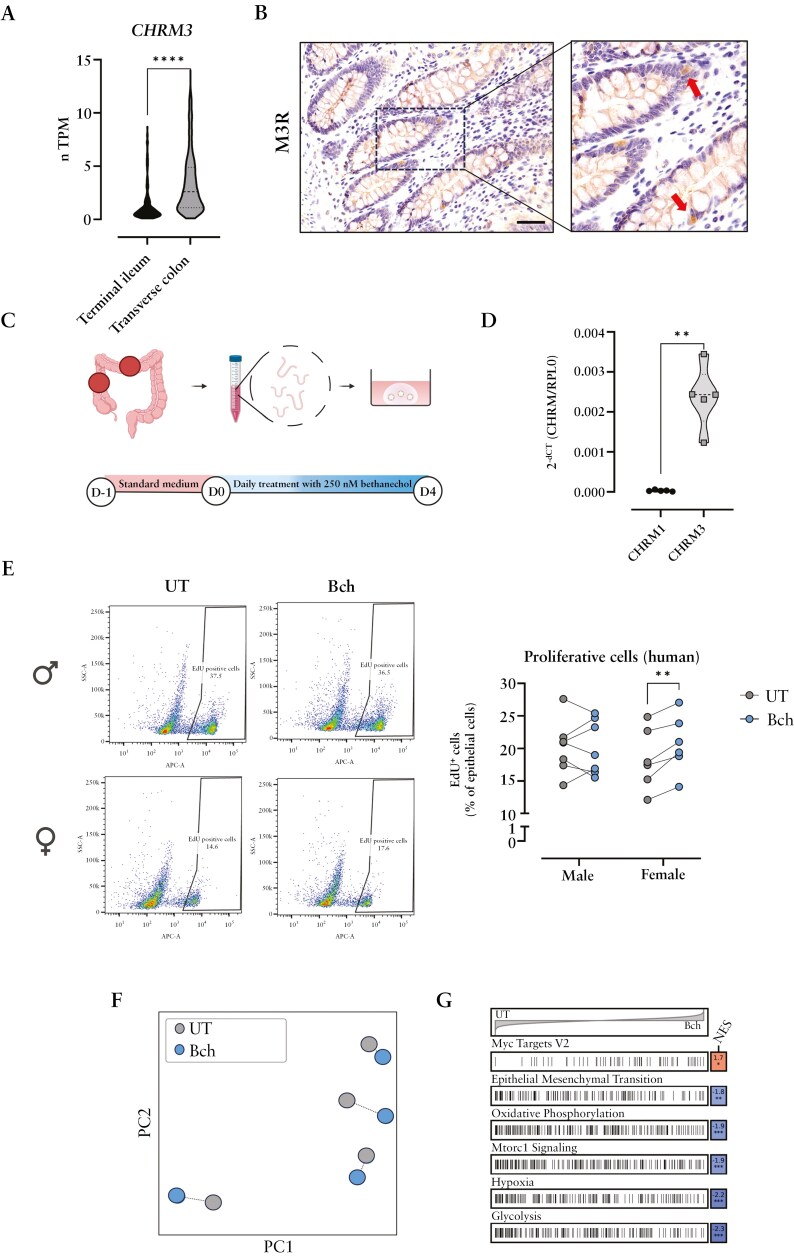
**Bethanechol treatment boosts the proliferative capacity of colonoids derived from human females.** (**a**) Violin box represents transcripts per million (nTPM) of *CHRM3* in terminal ileum and transverse colon. Data were obtained from proteinatlas.org. (**b**) Representative images of M3R immunostaining of colon with red arrows showing M3R-positive cells. Scale bar = 100 µm (20×). (**c**) Schematic representation of human colonic organoid generation. Crypts were isolated from biopsies collected from ascending and transverse colon and seeded in Matrigel. Organoids (≥ passage 3) were treated with 250 nM Bch on a daily basis after 24 hours incubation with standard medium. (**d**) mRNA expression of *CHRM1* and *CHRM3* was measured in human colonic organoids by qPCR. (**e**) EdU cell proliferation assay was performed in single cell suspension from UT and Bch-treated colonoids. (**f**) PCA of transformed count data from bulk RNA sequencing of UT colonoids and organoids treated with Bch. (**g**) Hallmark gene-set enrichment analysis of differential gene expression in UT and Bch-treated female colonoids. Statistical differences were detected by (**a**,**d**) Mann Whitney test or (**e**) multiple paired t tests. **P < .01, ****P < .0001. UT: untreated; Bch: bethanechol; NES = normalized enrichment score.

Next, we explored whether the sex-specific findings observed in murine organoids could be replicated in human colonic organoids. Human RNA seq-analysis revealed high expression of *CHRM3* in the colon compared to small intestine (proteinatlas.org) (Figure 5a). In line, we could confirm the epithelial presence of M3R in human colonic tissue sections (Figure 5b). Biopsies from ascending and transverse colon of male and female donors were used to generate colonoids for in vitro experiments (Figure 5c, Table 3). qPCR analysis of cultured organoids confirmed the prominent expression of *CHRM3* as compared to *CHRM1* (Figure 5d). Next, we investigated the effects of mAChR activation in human colonic organoids employing Bch treatment. Interestingly, similar to murine organoids, female-derived colonoids exhibited increased proliferation after 4 days of Bch treatment, while in male-derived organoids Bch had no effect on proliferation (Figure 5e). Bulk RNA sequencing analysis of Bch-treated female human colonoids subsequently confirmed pathway changes induced by Bch, such as the upregulation of Myc targets or downregulation of epithelial mesenchymal transition, oxidative phosphorylation, mTORC1 signaling, hypoxia, or glycolysis (Figure 5f,g). Interestingly, the downregulation of the pathway oxidative phosphorylation appeared similar to the effect of Bch on murine female Lgr5-EGFP + cells (Figure 4h). Conversely and in line with the murine data, no prominent alterations were observed in Bch-treated male human colonoids. Collectively, our data thus point at sex-specific differences in the modulation of IECs, and epithelial progenitor cell types in particular, by cholinergic signaling, which importantly appears to translate in part into human IECs.

**Table 3.** Clinical characteristics of donors for human organoid generation.

Total number of donors	13	
Sex	Male	Female
	7 (53.8)	6 (46.2)
**Age (mean ± SD)**	59.3 ± 12.2	45.3 ± 16.8
**Biopsy localization**		
Ascending colon	1 (14.3)	3 (50)
Transverse colon	6 (85.7)	3 (50)

Values are *n* (%) unless otherwise indicated.

## 4. Discussion

The ISC niche comprises a wide range of cells that release different modulators that play a decisive role in intestinal homeostasis. The impact of the prominent neuromodulator ACh is not confined to the ENS, but prominently extends to modulate epithelial functions.^5^ To investigate this in more detail, we here explored modulations of intestinal epithelial M3R signaling as well as cholinergic, muscarinic receptor agonism and its impact on colonic ISC homeostasis.

First, we demonstrated the presence of the M3R subtype in colonic Lgr5-EGFP + progenitor cells. Several studies reported similar findings in the murine small intestine.^9,10^ Nonetheless, studies focusing on the colonic compartment are scarce. Khan et al. indicated that mAChRs, located in the colonic epithelium, would regulate the response to intestinal inflammation.^17^ By utilizing the Vil.M3R mouse model, we here revealed that the epithelial M3R indeed appears to play opposite roles in male as compared to female mice, prominently altering the population of colonic *Lgr5*-expressing progenitor cells. Sex-specific variations are evident in the stem cell phenotype of several organs including the gut.^19,55,56^ Our data suggest that the decrease in *Lgr5*-expressing progenitor cells in the male colon upon genetic M3R deletion is testosterone-independent. In contrast, the observed expansion of *Lgr5*-expressing progenitor cells in adult female Vil.M3R tissues may be driven by estrogen, which is in line with previous studies showing that estrogen promotes stem cell proliferation and self-renewal.^55,57^ Berger et al. highlighted the presence of sexual dimorphism in the response of colonoids to pharmacological M3R modulation in the context of prenatal stress.^58^ Specifically, colonoids from male mice exposed to stress exhibited increased size when treated with an M3R inhibitor, while their female counterparts displayed the opposite.^58^

As a result of epithelial genetic M3R deletion, we observed the expansion of p-EGFR + tuft cells along with promoted production of colonic ACh. Tuft cells are uniquely equipped among IEC to produce ACh and may play a critical role in stemness regulation via the EGFR/Akt axis.^39^ M3R, in particular, was reported as a potential link between ACh and the activation of the EGFR/Akt pathway in gastric cancer cells.^59^ Despite the observation that male and female Vil.M3R mice displayed no changes in the expression of PI3K, divergent phenotypes were observed between males and females in downstream signaling of the PI3K/Akt pathway. These changes were mainly detected in tissues from male mice, which might indicate the involvement of female sex hormones in buffering the effect of PI3K/Akt activation on ISCs. Indeed, estrogen has been implicated in the regulation of intestinal homeostasis via the regulation of PI3K activity and Akt phosphorylation.^60^ Furthermore, Hedges et al. showed that pharmacological inhibition of PI3K induced sex-dependent effects on the expression of liver antioxidant genes.^61^

Importantly, we could observe a prominent sexual dimorphism in response to intestinal injury. Female mice with epithelial genetic M3R ablation appeared protected from the damaging effects of DSS, while males showed exacerbated inflammation. These findings could in part be explained by the observed variations in *Lgr5*-expressing progenitor cells between male and female Vil.M3R mice. The importance of Lgr5-EGFP + progenitor cells in epithelial regeneration and repair is well documented.^44^ Moreover, the observed response to injury may also involve an interplay with the sexual identity of the epithelium. For instance, Zitter et al. demonstrated that Lgr5-positive progenitor cells in males are more radiosensitive than in females,^62^ however non-treated sex-matched control mice were employed in our study to control for such potential effects. Another factor that might play a role in our in vivo model is a potential divergent cholinergic modulation of regeneration. Besides M3R, murine colonic IECs express M1R, which was reported to promote cell proliferation.^17^ Therefore, it can be hypothesized that M1R may potentially compensate the loss of M3R function in female colonic epithelium in vivo, thereby preserving the *Lgr5*-expressing progenitor cell compartment, but not in male colonic epithelium. These findings are corroborated by a previous study that demonstrated the efficacy of Bch in the absence of M3R.^12^

Intestinal organoids show transcriptional changes when compared to their tissue of origin, however, certain transcriptional programs from distinct compartments, including gender, also appear conserved.^63^ Indeed, sex-specific modulations of IEC homeostasis by cholinergic, muscarinic signaling appeared to translate into in vitro models, where we could observe an expansion of Lgr5-EGFP + cells and increased proliferation in female-derived colonoids treated with Bch. Metabolically, Bch treatment promoted lactate production in treated female-derived IECs in vitro. Levin et al. reported similar findings with modulation of glycolytic capacity by Bch in the rabbit urinary bladder.^64^ Since metabolic identity is a crucial factor influencing stemness and the proliferative potential of the intestinal epithelium,^52^ the increase in lactate is particularly significant as it has been shown to enhance the proliferation of Lgr5-positive progenitor cells.^65^ Moreover, in the same direction, bulk RNA sequencing analysis of Lgr5-EGFP + progenitor cells revealed differential regulation of certain metabolic pathways such as cholesterol homeostasis and mitochondrial OXPHOS in response to Bch treatment in female organoids. Upregulation of cholesterol synthesis is known to drive ISC proliferation.^66,67^ In addition, proliferative ISCs suppress OXPHOS by reducing the activity of the mitochondrial pyruvate carrier (MPC), which restricts the transport of pyruvate into the mitochondria and facilitates their proliferative state.^68^ Importantly, our RNA sequencing analysis revealed apparent transcriptional changes induced by Bch treatment in murine and human female samples, yet did not reveal prominent changes to investigated male samples, which in addition supports the observed biological sex-specific effects of Bch. Intriguingly, Bch treatment finally promoted proliferation and appeared to induce similar pathway changes in cultured female HIOs as observed in murine colonoids, which may support the translation of our results into human tissues at least in part.

In conclusion, our study highlights the critical role of cholinergic signaling, and epithelial M3R signaling in particular, in modulating colonic progenitor cells and IEC differentiation, revealing regional intestinal differences and significant sex-specific differences. Moreover, the observed pronounced differences in intestinal regeneration in Vil.M3R mice may suggest potential therapeutic applications for targeting M3R signaling in treating intestinal disorders in a sex-specific manner.

## Supplementary Material

jjaf038_suppl_Supplementary_Figure_1

## Data Availability

The data underlying this article are available in the article and its supplementary material. RNA Sequencing data have been deposited to Gene Expression Omnibus with the accession codes GSE286189 and GSE286190.

## Conference presentation

United European Gastroenterology (UEG) week 2023, Copenhagen, Denmark.

## Data transparency

Data and materials described in this study will be made available upon request.
