# Supplementary material for: Epithelial genetic muscarinic receptor 3 ablation induces sex-specific modulation of colonic intestinal progenitor cells and response to intestinal injury
Source: J Crohns Colitis. 2025 Mar 7;19(6):jjaf038. doi: 10.1093/ecco-jcc/jjaf038 (PMC12138776; doi:10.1093/ecco-jcc/jjaf038)
Supplement: jjaf038_suppl_Supplementary_Figure_1 [file jjaf038_suppl_supplementary_figure_1.pdf]

Supplementary Figures

Figures legends

Figure S1. Levels of sex hormones in adult and juvenile Vil.M3R mice. Serum levels of (**a**) testosterone and (**b**) estradiol were measured by ELISA in adult and 22-days old Vil.M3R mice. Statistical differences were detected by Mann Whitney test. All results are shown as truncated violin plots with median represented as dashed line. *$p < 0.05$, **$p < 0.01$.

Figure S2. Effect of epithelial M3R ablation on tuft cell differentiation. Representative images of DCLK1 immunostaining of colon harvested from WT and Vil.M3R mice with red arrows showing DCLK1-positive cells. Scale bar = 50 µm (20x). DCLK1-positive tuft cells were counted along colonic crypts. Results are shown as truncated violin plots with median represented as dashed line. Statistical differences were detected by 2way ANOVA with Šídák's multiple comparisons test. **$p < 0.01$.

Figure S3. Effect of M3R ablation on progenitor cells on tuft cell expansion. (**a**) Colonic p-EGFR-positive cells were counted in Lgr5-EGFP-IRES-CreERT2 × M3R fl/fl mice 5 days after tamoxifen induction. Negative control was included without primary antibody. Scale bar = 50 µm (20x). (**b**) M3R was ablated in Prox1-positive progenitors by tamoxifen induction of Prox1-CreERT2 × M3R fl/fl mice. Numbers of colonic p-EGFR-positive cells were assessed in Prox1-M3R +/+ and Prox1-M3R -/- after 5 days of induction. Scale bar = 50 µm (20x). Data are shown as truncated violin plots with median represented as dashed line.

Fig. S1

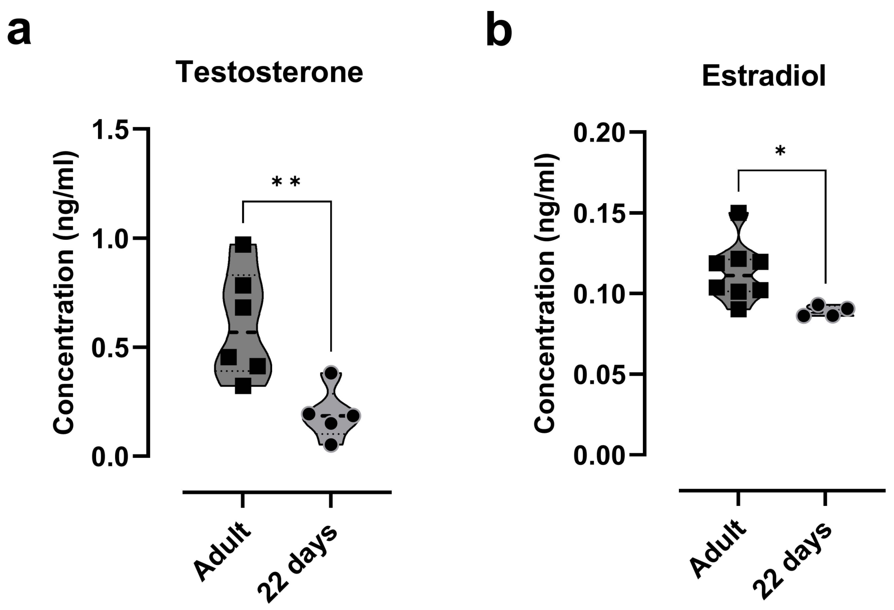
a
Testosterone
Concentration (ng/ml)
1.5
1.0
0.5
0.0
**
Adult
22 days
b
Estradiol
Concentration (ng/ml)
0.20
0.15
0.10
0.05
0.00
*
Adult
22 days

Fig. S2

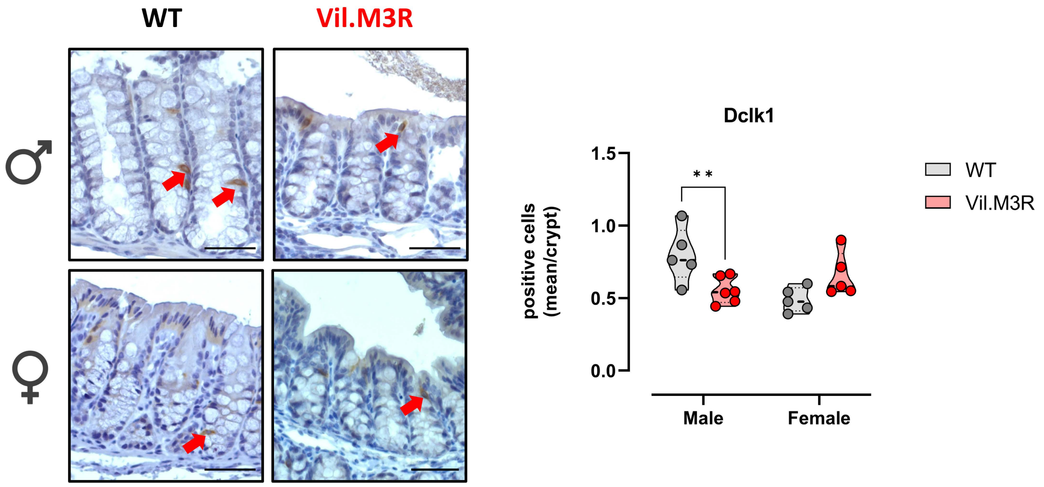

WT
Vil.M3R
♂
♀
Dclk1
1.5
1.0
0.5
0.0
positive cells
(mean/crypt)
**
Male
Female
WT
Vil.M3R

Fig. S3

a

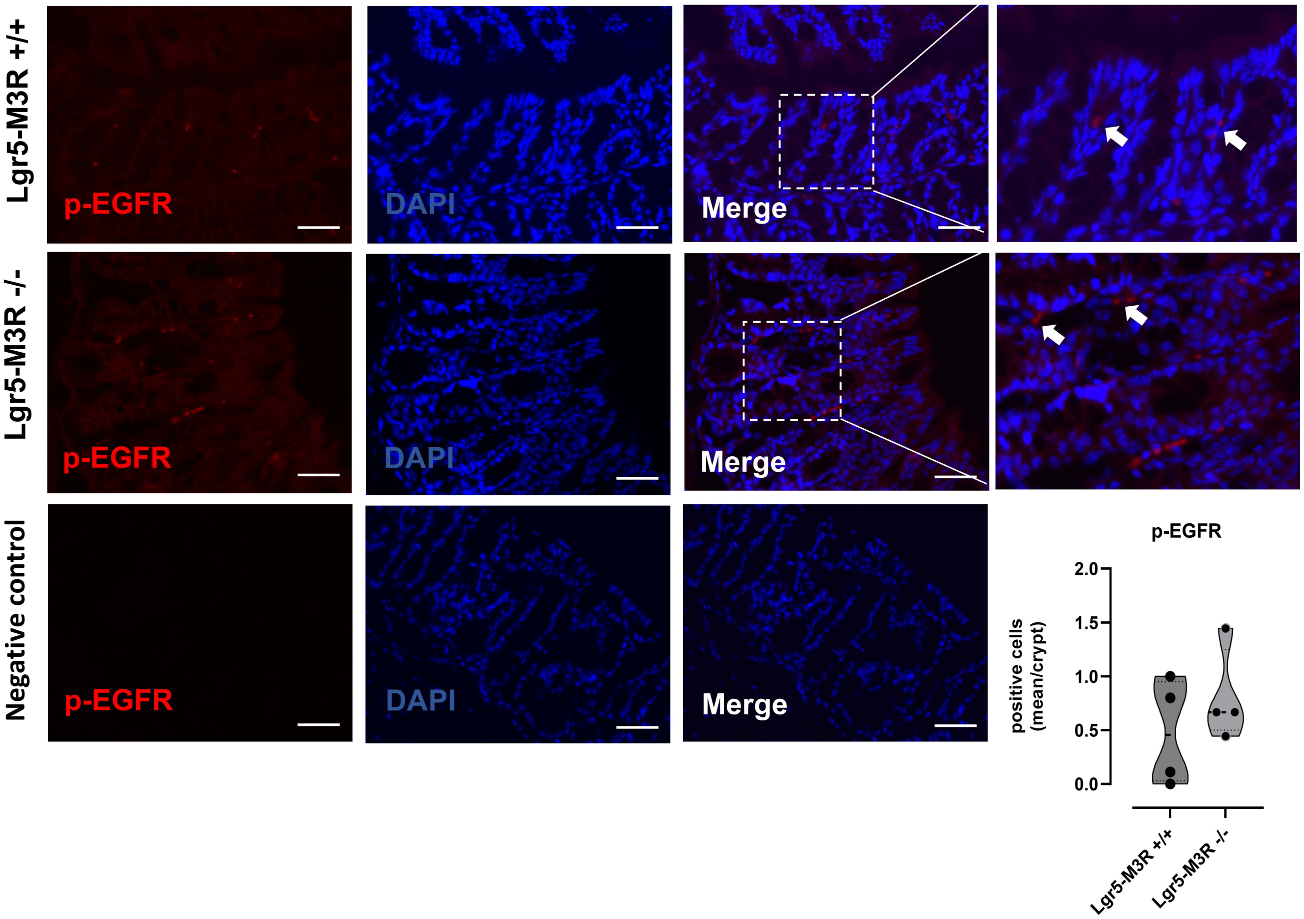
Lgr5-M3R +/+
Lgr5-M3R -/-
Negative control
p-EGFR
DAPI
Merge
p-EGFR
positive cells (mean/crypt)
2.0
1.5
1.0
0.5
0.0
Lgr5-M3R +/+
Lgr5-M3R -/-

b

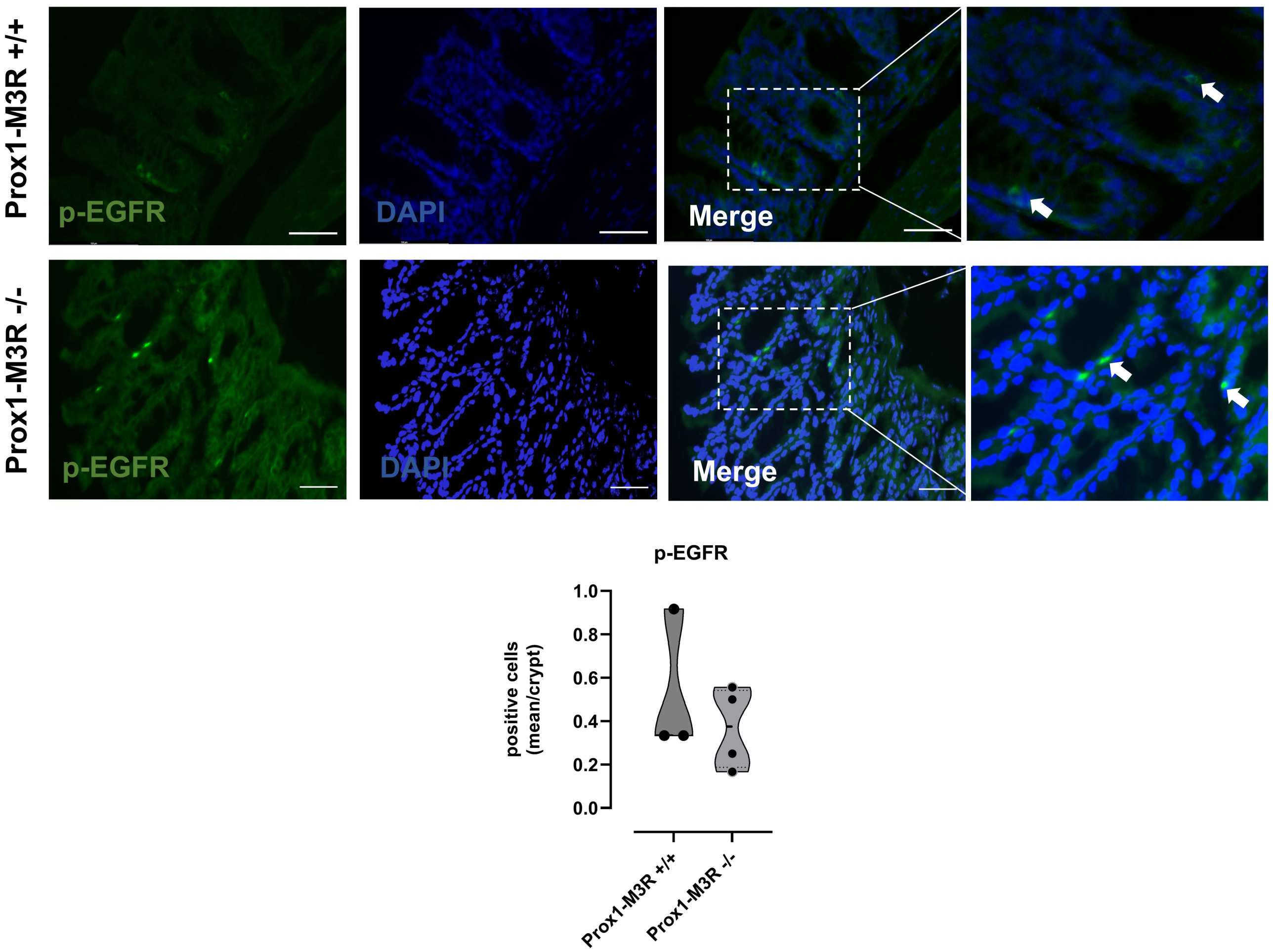
Prox1-M3R +/+
Prox1-M3R -/-
p-EGFR
DAPI
Merge
p-EGFR
positive cells (mean/crypt)
1.0
0.8
0.6
0.4
0.2
0.0
Prox1-M3R +/+
Prox1-M3R -/-
